# Dam-mediated flooding impact on outpatient attendance and diarrhoea cases in northern Ghana: a mixed methods study

**DOI:** 10.1186/s12889-022-14568-w

**Published:** 2022-11-17

**Authors:** Winfred Dotse-Gborgbortsi, Duah Dwomoh, Moses Asamoah, Faustina Twumwaa Gyimah, Mawuli Dzodzomenyo, Chengxiu Li, George Akowuah, Anthony Ofosu, Jim Wright

**Affiliations:** 1 School of Geography and Environmental Science, University of Southampton, Highfield, Southampton, SO17 1BJ UK; 2 WorldPop, School of Geography and Environmental Science, University of Southampton, Southampton, UK; 3 Ghana School of Public Health, University of Ghana, P.O. Box LG 13, Legon, Accra, Ghana; 4 Institute of Statistical Social and Economic Research, University of Ghana, P.O. Box LG 74, Legon, Accra, Ghana; 5 Upper East Regional Health Directorate, Ghana Health Service, Bolgatanga, Ghana; 6 National Headquarters, Ghana Health Services, Accra, Ghana

**Keywords:** Floods, Climate, Health information systems, Geographic information systems, Diarrhoea

## Abstract

**Background:**

Floods are the most frequently occurring natural disaster and constitute a significant public health risk. Several operational satellite-based flood detection systems quantify flooding extent, but it is unclear how far the choice of satellite-based flood product affects the findings of epidemiological studies of associated public health risks. Few studies of flooding’s health impacts have used mixed methods to enrich understanding of these impacts. This study therefore aims to evaluate the relationship between two satellite-derived flood products with outpatient attendance and diarrhoeal disease in northern Ghana, identifying plausible reasons for observed relationships via qualitative interviews.

**Methods:**

A convergent parallel mixed methods design combined an ecological time series with focus group discussions and key informant interviews. Through an ecological time series component, monthly outpatient attendance and diarrhoea case counts from health facilities in two flood-prone districts for 2016–2020 were integrated with monthly flooding map layers classified via the Moderate Resolution Imaging Spectroradiometer (MODIS) and Landsat satellite sensors. The relationship between reported diarrhoea and outpatient attendance with flooding was examined using Poisson regression, controlling for seasonality and facility catchment population. Four focus group discussions with affected community members and four key informant interviews with health professionals explored flooding’s impact on healthcare delivery and access.

**Results:**

Flooding detected via Landsat better predicted outpatient attendance and diarrhoea than flooding via MODIS. Outpatient attendance significantly reduced as LandSat-derived flood area per facility catchment increased (adjusted Incidence Rate Ratio = 0.78, 95% CI: 0.61–0.99, *p* < 0.05), whilst reported diarrhoea significantly increased with flood area per facility catchment (adjusted Incidence Rate Ratio = 4.27, 95% CI: 2.74—6.63, *p* < 0.001). Key informants noted how flooding affected access to health services as patients and health professionals could not reach the health facility and emergency referrals were unable to travel.

**Conclusions:**

The significant reduction in outpatient attendance during flooding suggests that flooding impairs healthcare delivery. The relationship is sensitive to the choice of satellite-derived flood product, so future studies should consider integrating multiple sources of satellite imagery for more robust exposure assessment. Health teams and communities should plan spatially targeted flood mitigation and health system adaptation strategies that explicitly address population and workforce mobility issues.

**Supplementary Information:**

The online version contains supplementary material available at 10.1186/s12889-022-14568-w.

## Introduction

Floods are the most frequently occurring form of natural disaster [1], affecting an estimated 2.8 billion people between 1980 and 2013 [2]. Floods are predicted to become more frequent in smaller catchments globally given more intense rainfall events. Population exposure to flooding is predicted to increase, particularly in Asia, Africa, Central and South America as intense tropical cyclones become more frequent [3]. With 37,600 dams higher than 15 m worldwide in 2014 and a further 3,700 planned or under construction [4], fluvial flood regimes (where rivers overflow their banks) are increasingly mediated by dams. Whilst some dams are constructed explicitly to mitigate flood risk [5], the effects of the majority are more complex and may profoundly affect the livelihoods of downstream populations [6]. Given this context, it is important to understand their impacts on public health, so as to inform flood preparedness and mitigation efforts, particularly downstream of dams.

Flooding has often been found to increase diarrhoeal disease risk alongside that for other waterborne diseases, but there is less evidence on its impact on healthcare utilisation. In a systematic review of flooding and diarrhoea disease risk, 19 out of 25 quantitative analyses of the relationship reported a significant positive association, with plausible dose–response relationships observed in several studies [7]. Positive relationships have been identified in studies in low and middle income countries for cholera, rotavirus, cryptosporidiosis, but also diarrhoea not attributable to a specific pathogen [8]. Many water-borne disease outbreaks associated with extreme weather events such as flooding result in deaths [9]. Flooding can mobilise pathogens in soils, animal or human faeces, and sediments, with pathogens associated with resuspended sediments. For example, increased concentrations of enteric viruses have been observed in surface waters during extreme flood events [10]. Flooding can also contaminate groundwaters, both directly and as the subsurface becomes saturated, facilitating pathogen transport [7]. Alongside greater potential for food contamination during floods, sanitation and water infra-structure may also be compromised by flooding, with backflows contaminating water systems. Through disruption to travel, flooding may also affect health facility utilisation. However, despite several studies of diarrhoea risk from flooding relying on outpatient records [11, 12], no studies of flooding’s impact on attendance for routine or preventative healthcare were identified in a recent systematic review [13]. Subsequently, a Cambodian study found that flooding had no impact on attendance for childbirth and a moderate impact on outpatient attendance in some districts only [14].

For ecological studies seeking to quantify health risks from flooding, a key issue is how to assess population flood exposure status. One approach is to use time series of flood imagery from satellite remote sensing for exposure assessment. Several such data sets are routinely produced, including daily near-real time global flood mapping from the Moderate Resolution Imaging Spectroradiometer (MODIS) satellite sensor [15]. Whilst there is general agreement between these different products for large flood events, detailed spatio-temporal patterns vary in data-sparse regions such as Africa [16]. The impact of MODIS-derived flooding on health outcomes and healthcare utilisation has previously been assessed in Cambodia [14], but it is unclear whether the observed strength of relationships could be affected by the choice of flood data product. Although flood exposure misclassification has attracted little attention in related systematic reviews [13], it has consequent potential implications for quantifying health risks associated with flooding via epidemiological studies.

A systematic mapping of flooding’s health impacts [17] also identified a paucity of mixed methods studies, recommending that such studies be used to deepen understanding of impacts. Although there have subsequently been mixed methods studies of flooding’s impacts on integrated community case management in Bangladesh [18] and its long-term wellbeing impacts in the UK [19], such studies remain scarce.

In northern Ghana, rainfall is associated with diarrhoea in sludge applying communities [20], implying that floods could have a greater impact. Furthermore, extreme weather events limit client’s ability to reach health facilities in urban northern Ghana [21] but the perception of service providers and their capacity to render services in floods is less understood. There is a lack of studies linking dam-mediated flood events and routine health data with contextual explanations from the communities impacted. Thus, observing flood events from satellite imagery in the study area [22], and the availability of routine health data provides the opportunity for a mixed methods study.

The aim of this study is therefore firstly to assess the relationship between dam-mediated flooding as detected via the MODIS versus Landsat satellite sensors and reported outpatient attendance at health facilities. Secondly, we aim to assess the effect of dam-mediated flooding as detected via the two sensors on monthly acute diarrhoea cases reported by health facilities. Thirdly, via qualitative fieldwork, we aim to explain flooding’s impact on healthcare provision and utilisation. In doing so, we aim to develop a methodology that integrates remotely sensed data concerning floods, routinely collected outpatient data, and related geospatial data on attending populations and travel.

## Methodology

### Overview

This study used a convergent parallel mixed methods design approach that combined an ecological time series with focus group discussions and key informant interviews. Through a quantitative, ecological study component, we examined the relationship between outpatient attendance and acute watery diarrhoea (reported monthly by health facilities) with dam-mediated flooding, whilst controlling for rainfall. We use two satellite-derived flood data products to quantify flood-affected area and population to examine how the choice of flood exposure metric affects the strength of relationship with healthcare utilisation and diarrhoea. Through Focus Group Discussions (FGDs) and Key Informant Interviews (KIIs), the qualitative component examined flood impacts on healthcare delivery, access and health outcomes from the perspectives of flood-affected communities and healthcare workers.

### Setting

The study area comprises Savelugu district in Northern Region and Talensi district in Upper East Region. Talensi had a population of 87,021 in 2021, whilst Savelugu’s population was 122,888 [23]. Both districts are affected by widespread flooding from planned overspills from the Bagre Dam, a multi-purpose dam in Burkina Faso on the White Volta constructed in 1992 [24]. When the dam water reaches a critical level (235 m above sea level), typically following rains in August and September, its waters are released to minimise risk of dam wall collapse. Dam overspill has inundated downstream riverine communities in northern Ghana in every study year except 2017. Working with other institutions and non-governmental organisations, Ghana’s National Disaster Management Organisation (NADMO) issues advance warnings of dam releases, raising public awareness of flooding, and managing mitigation measures such as organising Disaster Volunteer Groups [25].

Both districts have benefitted from national initiatives to achieve universal health coverage. To address financial barriers to healthcare utilisation, Ghana operates a National Health Insurance Scheme, in which 73% of its population were enrolled nationally by 2017 [26]. The implementation of a Community-based Health Planning and Services (CHPS) initiative has densified the network of primary care facilities and overcome geographic barriers to healthcare utilisation, though issues such as variable healthcare quality remain problematic [27].

### Secondary data sources

#### Diarrhoea disease and outpatient data

Monthly counts of outpatients attending primary and secondary healthcare facilities, together with monthly reported cases of acute watery diarrhoea, were obtained at facility level from Ghana Health Services (GHS)’ District Health Management Information System II (DHIMS2) database [28], together with facility locations. We chose monthly Outpatient Department (OPD) attendance and diarrhoea case counts for 2016 to 2020 following consultation with GHS staff, reflecting greater reporting completeness from 2016 onwards. The impact of flooding on outpatient attendance was assessed to identify whether flooding reduced facility attendance for diarrhoea treatment through disruption to patient travel.

#### Satellite-derived flood imagery

Two satellite-derived flood imagery time series were collated for 2016–2020: the near real-time (NRT) Global MODIS Flood Mapping product and the Landsat-derived Global Surface Water (GSW) database [29]. The MODIS flood product is derived from a band ratio water detection algorithm [15]. The detected water is compared to a reference water layer that shows the extent of persistent water features, and any pixels found outside the persistent water extent are marked as flooded. The product is available globally at approximately 250 m spatial resolution with daily coverage. Multi-day composite products are available to minimize cloud cover issues including 2-day, 3-day, and 14-day composites [30]. We used 14-day composites to generate monthly maximum flood extent from 2016–2020 to align with outpatient reporting periods. To assess the sensitivity of findings to the choice of flood data product, monthly maximum flooding extent was also identified from the Landsat-derived GSW product [29]. This product comprises monthly map layers of permanent and seasonal surface water generated using Landsat 5, 7, and 8 imagery from 1984–2019 [29]. For flood exposure assessment, seasonal surface water areas were treated as flooded for 2016–2019.

#### Population counts

To model flood-affected populations and populations within each facility catchment, gridded population count estimates for 2010 and 2015 were downloaded from WorldPop at a resolution of 100 × 100 m. These estimates were generated via a random forest algorithm by combining map layers of human settlement patterns and land cover with areal population counts from the 2010 Ghanaian census [31].

#### Travel time model data

Land cover, elevation, road networks and water bodies were used to generate an impedance surface for modelling patient journey times to healthcare facilities. An impedance surface is a model that assigns time penalties to grid cells within a landscape to estimate travel times between origins and destinations. Road networks and water bodies (rivers and lakes) were obtained from the OpenStreetMap global database [32] to represent enablers and barriers to travel, respectively, in our travel time model. A digital elevation map layer, the Void-filled Shuttle Radar Topography Mission with a spatial resolution of 30 m, was used to account for terrain effects on patient travel, whilst a 100 m resolution layer, the Copernicus Global Land Cover types for 2019 [33], represented land cover effects on travel.

#### Rainfall

To control for rainfall’s effect on diarrhoea and outpatient attendance, a gridded rainfall dataset, the Climate Hazards Group InfraRed Precipitation with Station data (CHIRPS), was retrieved for 2016–2020 [34]. CHIRPS incorporates 0.05° resolution satellite imagery with in-situ meteorological data to create a gridded daily rainfall time series.

### Secondary data preparation and integration

Patient travel to the nearest health facility was modelled via the impedance surface within ArcGIS Desktop 10.8, assuming patients walked to the nearest road and then travelled by motorised transport, following previous patient travel modelling studies in Ghana [35]. The effect of terrain on walking speed was modelled via Tobler’s hiking function [36]. Motorised travel speeds were based on maximum speed limits of 90 km/hr, 50 km/hr, and 30 km/hr for primary, secondary and tertiary roads respectively. The secondary and primary healthcare facility that lay closest to each grid cell in terms of modelled travel time was separately identified and used to represent facility catchments. Catchment boundaries were overlaid on monthly rainfall estimates and gridded population data, linearly projecting population counts between 2010 and 2020. For each facility, the monthly proportion of its catchment area and population affected by flooding was calculated, using both the Landsat GSW and NRT MODIS data products. Monthly facility-level data concerning flooding and rainfall were then integrated with DHIMS2 records. Since the GHS database does not differentiate zero case counts from nulls, null monthly case counts were treated as zeros where a health facility had returned outpatient attendance figures in that month.

### Key informant interviews and focus group discussions

A phenomenological approach was adopted for the qualitative study phase. Two researchers, MA (male) and FTG (female), with substantial qualitative research experience, and based outside the White Volta catchment, facilitated the FGDs and KIIs. FGD participants were flood-prone community members not previously known to the facilitator. The flood-affected communities were identified via satellite imagery and purposively selected after visiting the district NADMO office to validate the satellite observations and discuss the feasibility of interviews. Based on a maximum variation sampling strategy, residents of four flood-affected communities were selected via referral through a local NADMO officer and assembly member (a local elected representative). All participants were adults at least 18 years old and resident in the community for at least ten years and thus with experience of flooding. Participants were selected to represent community leaders (district assembly members or chiefs), community opinion leaders (i.e. influential figures such as community health volunteers, the highly educated such as teachers or nurses, agricultural leaders, or female trade association heads), or household heads.

Participants were recruited face-to-face and asked to identify other suitable participants. Four FGDs were conducted in Frafra, Dagbani or English, with eight individuals in each group, separated by gender to moderate power and so allow more open expression of opinions by participants [37]. Only invited participants attended the FGDs, which lasted an hour and took place on 16th and 21st September 2020 (following spilling of the Bagre Dam on 10^th^ August 2020) at Savelugu and Talensi. The discussion reached saturation on the fourth FGD. Each focus group discussed flooding history, impacts on health and healthcare utilisation and related coping strategies (see Additional file 1). The group met at a local government office and communicated in Dagbani (Savelugu) and Frafra (Talensi).

For KIIs, two respondents were selected from each district health management team. We used a purposive homogenous sampling strategy for KIIs, selecting participants because of their district healthcare roles. Interviews were conducted on the 17^th^ and 22^nd^ September 2020 at Savelugu and Talensi respectively. Face-to-face interviews in English lasted 45 min in participants’ offices using a mixture of semi-structured and open-ended questions. The interviewers had no previous relationship with respondents. Interview sessions followed a topic guide (see Additional file 2) that included questions on healthcare delivery and access, flood mitigation interventions, and health promotion issues in their respective districts. All FGD and KII interviews were audio-recorded, transcribed and field notes taken. No participant declined to participate in both FGD and KII.

### Data analysis

Facility-level analyses were conducted using random effects Poisson regression with robust standard errors that adjusted for seasonality and the logged total catchment population for each health facility. All the models adjusted for a month-year time interval to control for both seasonality and long-term trend in the time series data. We fitted separate models for flooding detected via LandSat versus via MODIS. Successive models of monthly outpatient attendance assessed the effects of mean monthly precipitation and patient travel time per facility, percentage of facility catchment area flooded per month, and both flooding and patient times together. Similarly, consecutive models of monthly reported diarrhoea cases examined flood-affected population per catchment and mean precipitation, percentage flood-affected area per catchment, and both flood-affected area and flood-affected population together. We compared different Poisson regression models, using the Akaike Information Criterion (AIC). Statistical analyses were conducted using Stata MP version 16 [38] and a p-value less than 0.05 was considered statistically significant.

For the qualitative component, MA and FTG transcribed the recordings and translated interviews, comparing results for omissions and accuracy. Transcriptions of KIIs and FGDs were analysed inductively and deductively using a thematic and content analysis approach [39] with NVivo 12 [40]. MA and FTG created a codebook, based on the study questions, observations, field notes, and information from the transcripts. All transcripts were coded line by line, with both researchers comparing coding. Extra codes emerging from analysis were introduced to collect new data. Re-coding was performed until the final themes and sub-themes were produced (Table 1). KIIs and FGDs were triangulated. To assess the validity of responses, comparable themes and follow-ups were employed to improve the data's resilience. Via meetings in March 2022, findings were disseminated to stakeholders responsible for disaster management, health professionals, and community members including the participants for their feedback and input. Quantitative and qualitative findings were integrated during interpretation [41], triangulating quantitative findings with FGD and KII data sources to develop comprehensive understanding of flooding’s impacts via a mixed methods approach.

**Table 1:** Thematic framework

Themes	Sub-themes
Healthcare access	Access to healthcare facilities during flooding
	Access to healthcare professionals during flooding
	Inaccessible road network • Impediments to emergency referral • Mobility of healthcare providers • Transporting of logistics and medical supplies
	Flooding impacts on healthcare facilities
Healthcare delivery	Inadequate staffing levels in community health facilities
	Few healthcare facilities serve more communities
	Health-seeking behaviour change among some community members
	Greater uptake of traditional medicine (e.g. herbal treatments)
Water, Sanitation, and Hygiene	Poor sanitation practice
	Impacts on community drinking water sources

## Results

### Trends in outpatient attendance and reported diarrhoea cases

The study analysed routine health data from 54 health facilities, 21 in Savelugu and 33 in Talensi districts. Most health facilities provided primary care (41 CHPS compounds or clinics; 11 health centres) with one public hospital in each district providing secondary care. In total, 1329 null monthly reports were excluded from the analysis, of which 660 monthly reports were from 11 CHPS facilities that never returned monthly reports. However, a further 488 null reports were considered as zero diarrhoea cases because there were outpatient cases within those reporting periods. There were 1,423 records (health facility-month-years) with diarrhoea cases. Diarrhoea reporting trends at facility level, including patterns of null reports, are further visualised in Additional file 3.

**Fig. 1:**
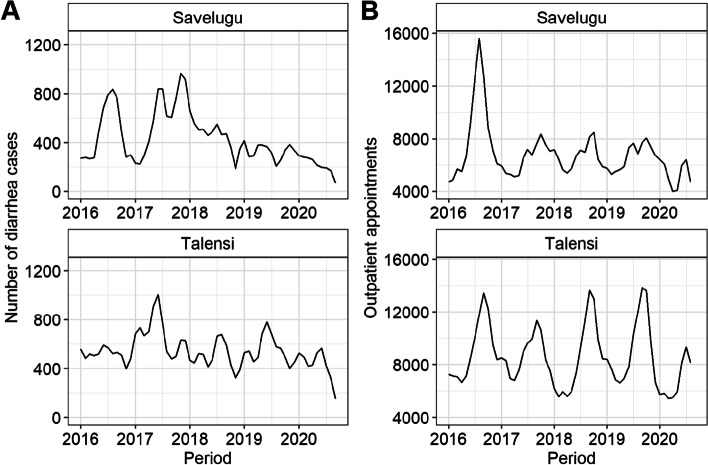
Trends in **a** reported diarrhoea cases and **b** outpatient attendance for Savelugu and Talensi districts, Ghana, 2016–2020

Between January 2016 and December 2020, there were a total of 216 diarrhoea cases per 1000 population (6.4% of all outpatient cases). A monthly median four diarrhoea cases per 1000 population was recorded over the period (minimum = 2, maximum = 8). On average, each person in the study area had 3.4 appointments for any health condition within the five year period. OPD attendance peaked seasonally during August–September, but there was no obvious seasonal pattern to reported diarrhoea cases as seen in Fig. 1. Outpatient attendance was high in 2016 in Savelugu, whilst diarrhoea cases peaked in 2016 and 2017 in Savelugu and 2017 in Talensi (Fig. 1). Monthly outpatient attendance and reported diarrhoea trends by health facility are displayed in Additional file 3.

### Trends in flooding patterns and rainfall

**Fig. 2:**
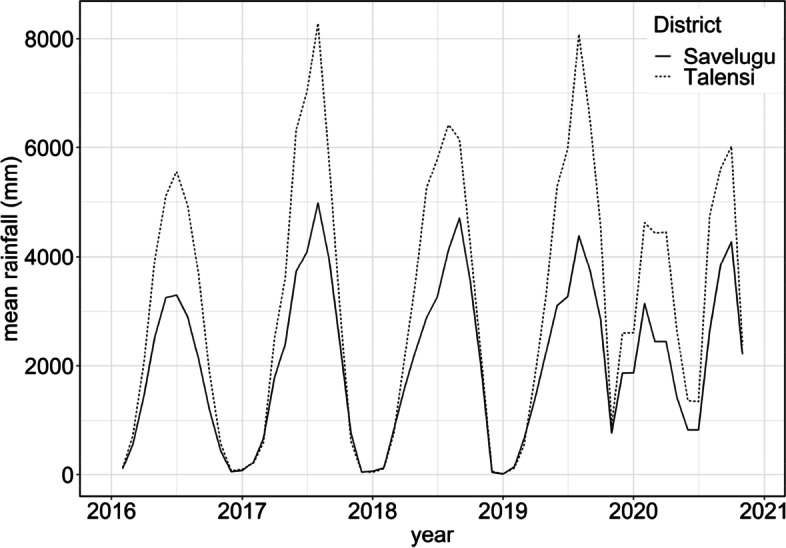
Monthly rainfall estimated via CHIRPS product from 2016 to 2020 in Savelugu and Talensi Districts, Ghana

**Fig. 3:**
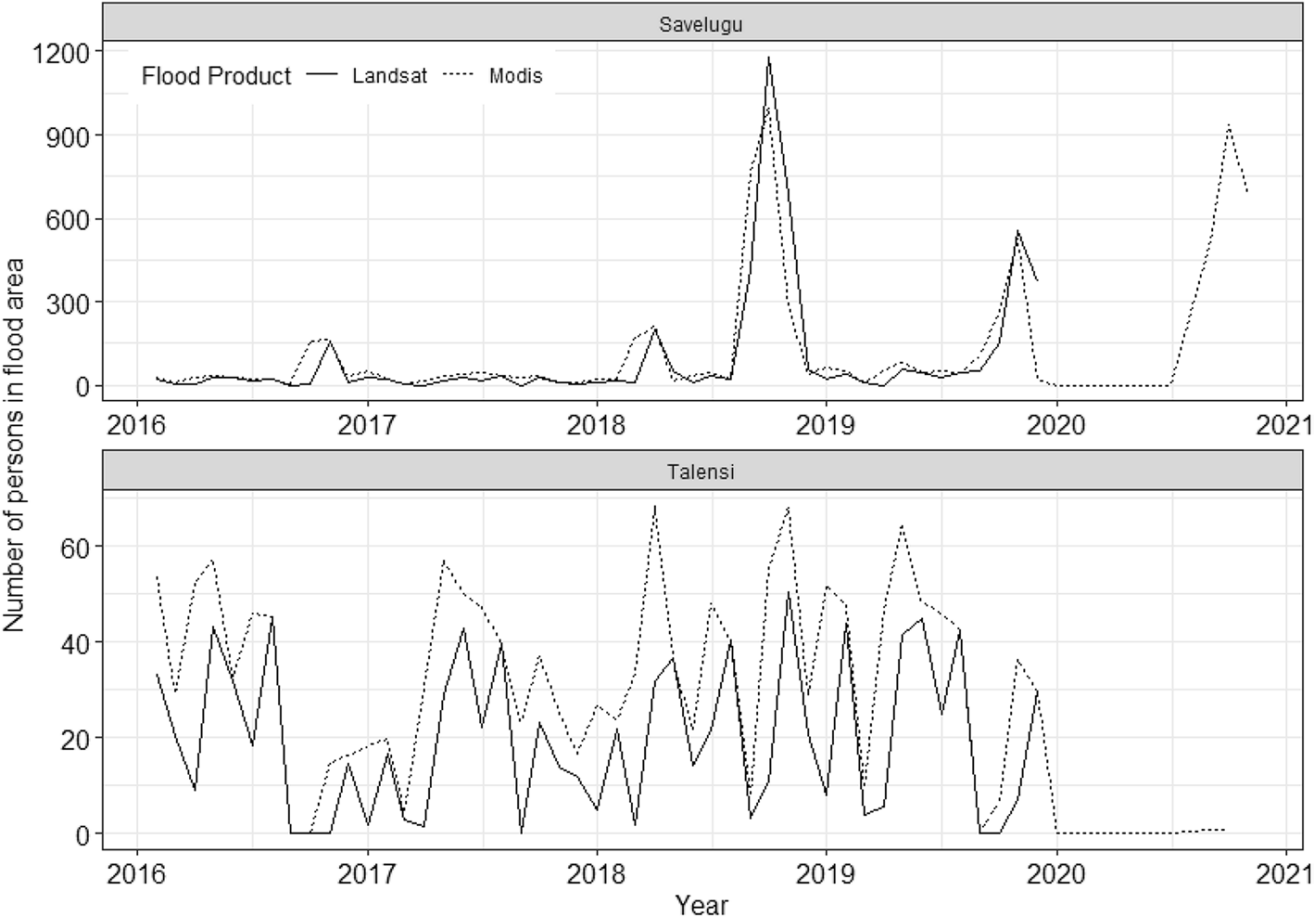
Population resident in inundated areas estimated via LandSat and MODIS-derived flood products from 2016 to 2020 in Savelugu and Talensi Districts, Ghana

Figure 2 shows more rainfall was recorded in Talensi compared to Savelugu. Although monthly peak rainfall was lower in 2020, minimum monthly rainfall was greater than in earlier years at approximately 1000 mm. Figure 3 shows that the estimated population exposed to flooding was broadly comparable for LandSat GSW compared to MODIS, with both suggesting the greatest population exposure was in Savelugu from 2018 onwards.

**Fig. 4:**
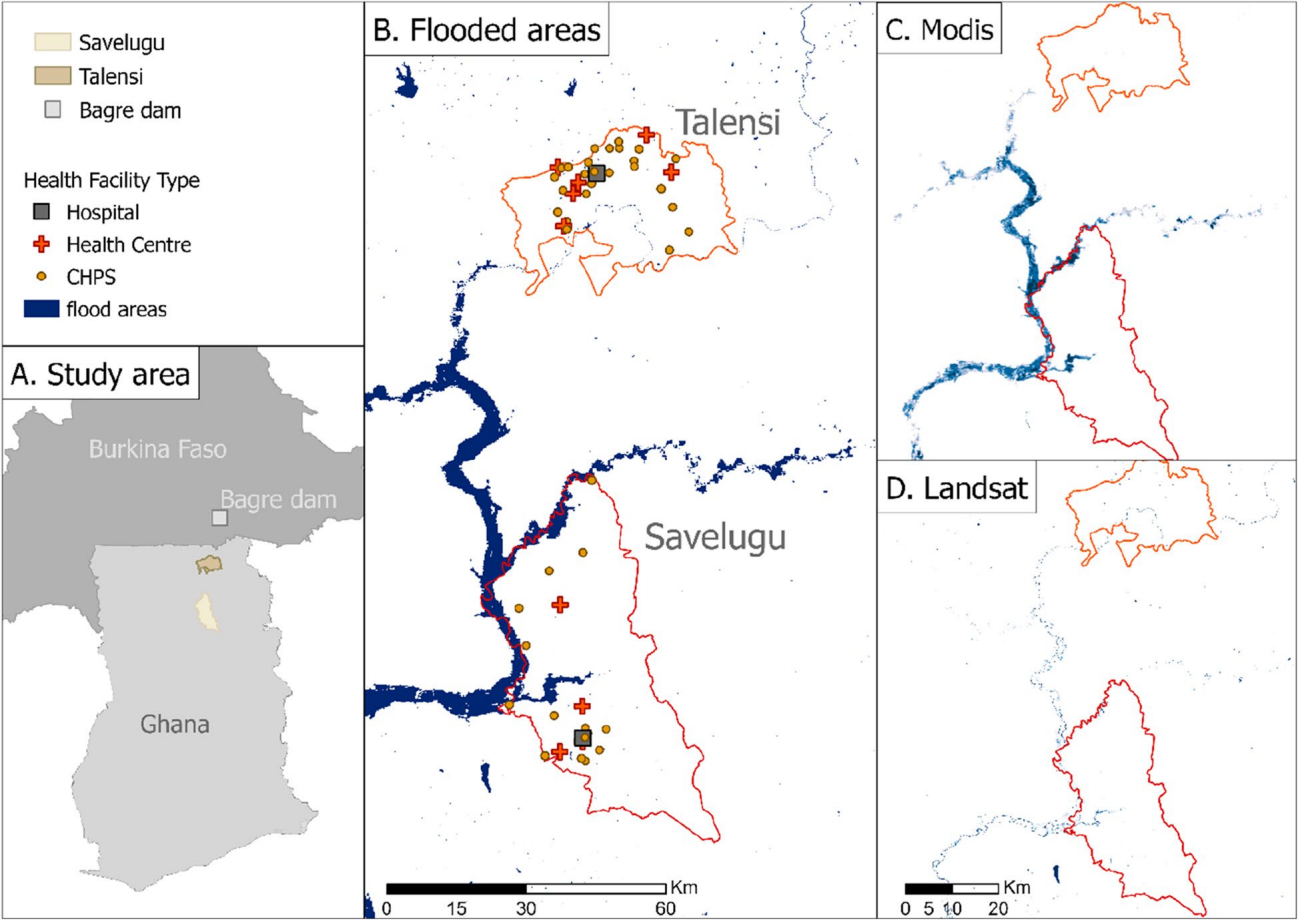
Map of health facilities in relation to maximum flood extent for 2016–2020, showing **a** locations of Savelugu and Talensi Districts relative to the Bagre Dam; **b** Flooding detected via either sensor **c** Flooding as detected via MODIS **d** Flooding as detected via Landsat

Figure 4 no facilities lay directly within flooded areas, but two of 54 facilities (4%) and 15 of 54 facilities (28%) were within 1 km of MODIS-derived and LandSat-derived flooding respectively. Additional file 3 displays the monthly flooding patterns per facility as detected via MODIS and LandSat.

### Effect of flooding on outpatient attendance

As shown in Table 2, the Landsat-based estimate (Model 4) of percentage flooded area per facility catchment better predicted OPD attendance than the MODIS-based model (Model 5) (AIC = 579,883.7 and 693,203.7 respectively). Model 4 estimates are presented in Table 3.

**Table 2:** Measures of performance for five models of monthly outpatient attendance at health facilities in Savelugu and Talensi districts, Ghana

**Model specification**	AIC	BIC
**Model 1:** mean travel time to health facility + mean monthly precipitation per facility catchment, controlling for total population per health facility catchment and seasonality	692,590.0	692,672.4
**Model 2:** Percentage of facility catchment area flooded (based on MODIS), controlling for total population per health facility catchment and seasonality	1,234,578.0	1,234,655.0
**Model 3**: Percentage of facility catchment area flooded (based on LandSat), controlling for health total facility catchment population and seasonality	1,099,016.0	1,099,090.0
**Model 4**: Percentage of facility catchment area flooded (based on LandSat) + mean travel time to health facility, controlling for total population per health facility catchment and seasonality	579,883.7	579,962.8
**Model 5:** Percentage of facility catchment area flooded (based on MODIS) + mean travel time to health facility, controlling for total population per health facility catchment and seasonality	693,203.7	693,286.1

**Table 3:** Adjusted incidence rate ratios derived from a random effects Poisson regression model of monthly outpatient attendance at health facilities in Savelugu and Talensi districts, Ghana

*Model coefficients*	*aIRR [95% CI]*
Percentage of facility catchment area covered by flooding based on the Landsat product	0.78 [0.61–0.99]*
Mean travel time to health facility (minutes)	0.97 [0.96–0.97]***

*P*-value notation: ***p < 0.001, **p < 0.01, *p < 0.05

*Abbreviation: aIRR* adjusted incidence rate

There was a statistically significant association between outpatient attendance and the percentage of each facility’s catchment area covered by flooding (Table 3). If the flooded area in a facility catchment increased by one percent, the outpatient attendance rate ratio would be expected to decrease to 78% (95% CI: 61%—99%, *p* < 0.05), controlling for a health facility’s catchment population and holding other variables constant. Similarly, a minute’s increase in travel time to the nearest health facility decreased the rate ratio of outpatient attendance 0.97 times (95% CI: 0.96 – 0.97, *p* < 0.001).

### Impact of flooding on facility-reported diarrhoea cases

When comparing regression model performance, the AIC results (Table 4) showed that using percentage of flooded area and flood-affected population per facility catchment based on Landsat predicted monthly diarrhoea cases better than the other five models (AIC = 115,512.5).

**Table 4:** Measures of performance for six models of monthly diarrhoea cases reported by health facilities in Savelugu and Talensi districts, Ghana

**Model specification**	AIC	BIC
**Model 1**: Monthly flood-affected population based on the MODIS product + average monthly precipitation, controlling for total population per health facility catchment and seasonality	148,352.3	148,435.3
**Model 2:** Monthly flood-affected population based on the LandSat product + average monthly precipitation, controlling for total population per health facility catchment and seasonality	117,561.1	117,640.3
**Model 3**: Percentage of flooding in facility catchment area based on the MODIS product, controlling for total population per health facility catchment and seasonality	155,344.2	155,421.7
**Model 4**: Percentage of flooding in facility catchment area based on the LandSat product, controlling for total population per health facility catchment and seasonality	140,473.2	140,547.1
**Model 5**: Monthly flood-affected population based on the LandSat product + Percentage of flooding in facility catchment area based on the LandSat product, controlling for total population per health facility catchment and seasonality	115,512.5	115,591.7
**Model 6**: Monthly flood-affected population based on the MODIS product + Percentage of flooding in facility catchment area based on the MODIS product, controlling for total population per health facility catchment and seasonality	148,362.4	148,445.4

As shown in Table 5, the percentage of flooded area per facility catchment and monthly flood-affected population were both significantly associated with reported diarrhoea cases. For each additional person inundated by floods, reported diarrhoea cases were expected to decrease 0.95 times (95% CI: 9.94 – 0.96, *p* < 0.001). In contrast, when the flooded area in a facility catchment increased by one percent, the reported diarrhoea rate ratio would be expected to increase 4.27 times (95% CI: 2.74 – 6.63, *p* < 0.001), controlling for each health facility’s catchment population and seasonality.

**Table 5:** Adjusted incidence rate ratios derived from a random effects Poisson regression model of monthly diarrhoea cases reported by health facilities in Savelugu and Talensi districts, Ghana

*Model coefficients*	aIRR [95% CI]
Monthly flood-affected population based on the LandSat product	0.95 [0.94–0.96]***
Percentage of flooding in facility catchment area based on the LandSat product	4.27 [2.74–6.63]***

*P*-value notation: ***p < 0.001, **p < 0.01, *p < 0.05

*Notation: aIRR* adjusted incidence rate ratio

### Healthcare access and health impacts reported via qualitative interviews and discussions

There was consensus that healthcare access was restricted during flooding for both staff and patients. A related theme emerged reflecting challenges with health service provision (Table 1). Sub-themes reflect inaccessible health facilities during flooding for both residents and healthcare workers. Particularly, health professionals in both districts recounted how their staff struggled with referrals and travel to facilities in cut-off communities. They reported their inability to conduct outreach services for vaccination, growth monitoring and follow-up home visits., with some staff using canoes to reach health facilities during flooding. Community respondents suggested that some health workers resided in towns beyond floodplains to avoid flooding impacts. Consequently, some health professionals who commuted from nearby towns were absent from facilities for up to a week, only resuming work when floodwaters receded. They described how limited healthcare access during flood season was exacerbated by lower staffing levels given flood-related commuting disruption among health professionals, but also as more patients attended those few facilities that remained open during flooding. A health professional noted:

*“The health staff can’t access some communities and their health facilities. Sometimes even referring emergency cases to a hospital from smaller health centres becomes a problem” (KII, Health Professional, Female, Savelugu).*

Community members also explained how flooding limited healthcare access, leading some to seek alternative forms of healthcare, exposing some to complications arising from untreated health conditions, and placing others at risk when travelling to seek healthcare. For instance, a mother recounted losing her baby in flood waters through an accident whilst returning from a health facility. Furthermore, they recounted how referred patients unable to reach a hospital during flooding could develop complications or die. Community respondents described how some patients sought alternative treatment, such as home-based herbal remedies, because they could not reach a health facility:

*“We use herbal treatment (concoction) for some illness during this time since we cannot go to hospital when the whole place is flooded” (FGD, Participant 4, Female, Talensi).*

*“How to even get to the district hospital in times of emergency becomes an issue. During this time, people are cut off from the district hospitals and some die as a result” (FGD, Participant 1, Male, Talensi).*

A final theme concerned exacerbation of open defecation’s health impacts and disruption to water and sanitation access during flooding, with consequent increased diarrhoea risk. Both health professionals and community members described this pathway. Health professionals considered diarrhoea cases to be higher in flood season because flood waters transported faecal contamination from open defaecation sites to drinking water sources. Similarly, community members attributed the higher diarrhoea cases to consuming contaminated water. Although communities were aware of diarrhoea risks from open defaecation and inadequate solid waste management, they felt helpless and unable to address these risks because of inadequate sanitation and waste management facilities. They noted greater risks for multiple health outcomes, including for diarrhoeal disease:

*“Everywhere in our communities is now occupied by water. So, where we usually ease ourselves and dump our rubbish, it all gets collected back into our water bodies. These are our sources for drinking and doing all other household chores. So, when the flood comes, it takes all these things back into our water sources, and we go to drink them. We know of this, but we don't have an option” (FGD, Participant 3, Female, Savelugu).*

## Discussion

To our knowledge, our study is the first to explore how choice of satellite-based flood product affects analyses of flood impacts on healthcare utilisation and diarrhoea. For both the NRT MODIS and Landsat-derived flood products, we find a significant reduction in reported outpatient attendance during flooding (Tables 2 and 3). Qualitative interviews with health professionals in both districts suggest that this decline in outpatient attendance may partly have been associated with staff travel disruptions during flooding, limiting the number of outpatients seen during flood periods. Although flood-related disruption to patient travel to facilities has been reported via key informant interviews in urban Ghana [21], disrupted journeys by healthcare staff to rural facilities has not. Both KIIs and FGDs suggested some healthcare staff lived in towns distant from rural health facilities to avoid flooding at these rural sites. As a consequence, both patients and service providers were unable to access health facilities during flooding.

After controlling for seasonality, monthly reported diarrhoea cases significantly increased in relation to the proportion of flood-affected area per catchment (Table 5), suggesting a positive exposure–response relationship. This effect was however somewhat mediated by the flood-affected population within each catchment. Consistent with this finding, the majority of included studies in two systematic reviews of diarrhoea risk from flooding found a positive relationship [7, 13].Given the known risk of food and water contamination [42], alongside pathogen transport, population displacement inhibiting safe water access, and damage to water and sanitation infrastructure during flooding [7], the observed increase in reported diarrhoea cases during flood months is epidemiologically plausible.

Our study has implications for using satellite-derived flood products for health risk assessment. To date, few studies have used satellite remote sensing to assess flood exposure for health risk assessment. Studies of diarrhoea risk included in a systematic review [7] mostly used other methods to assess exposure, such as river levels [43]. Some subsequent studies of health impacts have used geospatial disaster databases [44] and NRT MODIS satellite imagery [14] to assess flood status. Satellite-derived flood maps constitute an objectively defined measure of flood exposure that is internationally consistent and available in data-sparse regions, which could thus address a known lack of comparable exposure assessment metrics in many studies of flooding’s health impacts [9]. However, our study suggests that the performance of models predicting healthcare utilisation and health outcomes is sensitive to the choice of satellite-derived flood product, since we find better performing models of both healthcare utilisation and reported diarrhoea based on a LandSat product compared to NRT MODIS (Tables 2 and 4). This likely reflects underlying differences in the two flood products, noted in other studies [16]. The finer spatial resolution of LandSat (30 × 30 m) relative to MODIS (300 × 300 m) could reduce mixed pixel problems (classification difficulties arising from different land surfaces being present in the same grid square), thereby enabling detection of smaller flooding patches. However, whilst MODIS has a two day revisit time (the time elapsed between repeat observations of the same area), Landsat 8 has a 16 day revisit time [16], so LandSat imagery may not capture peak flood extent and so under-estimate exposure. Both products are also subject to common limitations. Neither product measures floodwater depth, which is important for assessing impassability of roads [45] and thereby patient travel disruption. Both optical sensors are affected by cloud cover [46], which may lead to under-estimation of flood exposure. Since recent studies [47] have integrated LandSat with synthetic aperture radar imagery from the Sentinel-1 sensor to map flooding despite cloud cover, we suggest that this approach would be appropriate in future studies of flooding’s public health impacts in data-sparse regions.

Given increased dam construction worldwide [4], Our study also provides evidence of the healthcare utilisation impacts of dam-mediated flooding on downstream populations [6]. However, whilst flooding in northern Ghana has increased despite declining rainfall since 1980, since the dam releases are one of several interacting drivers of flooding such as land cover change [25], it is difficult to attribute impacts to the Bagre Dam release schedule. Nonetheless, a hydrological modelling study [48] suggested that dam release could exacerbate flooding, raising White Volta water levels by 75 cm at 100-150 km downstream of relative to levels without dam operation. Management of dam water release schedules involves trade-offs between different impacts of such operations. Typically, trade-offs between power generation and water availability for irrigation are modelled in planning dam release schedules [49], but the impacts on health outcomes and healthcare utilisation have not been quantified when assessing trade-offs. In principle, health or healthcare utilisation impacts could be incorporated into trade-off modelling for dam release schedule management, but our study suggests that establishing health risk attributable to dam release would be complex and highly uncertain, limiting usefulness of such an extended modelling framework.

Aside from the issues affecting flood exposure assessment via satellite remote sensing outlined above, our study is subject to several limitations. The coarse monthly temporal resolution of DHIS2 data could have limited our ability to detect flooding’s effect on outpatient attendance and diarrhoeal disease. Weekly health facility reports could have enabled shorter lagged effects to be detected, but weekly reports are seldom completed for routine outpatient attendance since notifiable disease reporting takes priority. Similarly, our study findings may be affected by the accuracy and completeness of DHIS2 reporting, although DHIS2 reporting completeness is high in Ghana [28]. Whilst total outpatient attendance counts have been previously used to assess flooding impacts on healthcare utilisation, the risk of some diseases included within these counts could increase during flooding. For example, alongside diarrhoea risk, injury risk could increase during flood events [13], resulting in potential under-estimation of reduced outpatient attendance from flooding. Previous studies have used health management information systems data for conditions unrelated to flooding alongside outpatient counts, most notably childbirth deliveries at facilities [14]. However, healthcare-seeking behaviour for childbirth may differ from that for other health conditions. In relating population exposure to flooding with facility-level outpatient data, we did not model by-passing of health facilities, which is widespread for mothers’ journeys to give birth elsewhere in Ghana [50]. Given its ecological design, our study did not consider household or individual-level characteristics such as water source type, sanitation access, and socio-economic status [8, 51] that could moderate diarrhoeal disease risk from flooding.

In principle, the methods and workflow in this study can be generalised and replicated in settings where health management information system data are available. It could also be applied to other health conditions affected by flooding and recorded via DHIS2, such as injuries, skin or respiratory infections, and malaria [13]. Since the District Health Information System (DHIS2) platform deployed in Ghana as DHIMS2 is used in 60 countries at national level and 14 at pilot stage or sub-nationally [52], potentially these countries could replicate our study’s workflow, since the flood and population products we used are also global. However, despite the widespread international coverage of DHIS2 and increasing completeness in many countries such as Ghana [28], data quality remains problematic, varying between countries [53] and between system components, with for example lower accuracy for acute respiratory infection data compared to antenatal data in Malawi [54].

### Future research

Future work could further explore the uncertainties affecting the two flood exposure metrics we used and examine the relationship between alternative flood exposure measures and healthcare utilisation or health outcomes. Firstly, we examined residential population exposure to flooding using the WorldPop gridded population map layer. However, the WorldPop surface is just one of several available modelled gridded population layers. Since the WorldPop and LandScan surfaces distribute more population onto floodplains, they generate a much higher estimate of flood-affected population than a third gridded population layer (HRSL), which assumes populations avoid floodplains [55]. Future studies should therefore assess residential population flood exposure using multiple gridded population surfaces. Secondly, we assessed inundation of healthcare facility sites since flood damage to facilities disrupts healthcare delivery [21, 56]. However, flooding also disrupts electricity supply with outages sometimes affecting sites beyond the inundated area [56]. There would thus be potential to use new night-time satellite remote sensing products that enable detection of power outages to represent flood impacts on healthcare delivery, such as NASA’s Black Marble Night-time Light product [57].

Our FGDs and KIIs suggest further pathways by which flooding affects health and healthcare utilisation, which we did not measure in our study. These pathways entail disruption to referrals, patient travel to facilities, and staff travel to facilities. All three could be represented via satellite-derived flood products and explored via future studies. A Mozambican study recently incorporated flood extent from NRT MODIS into impedance surfaces, enabling modelling of daily variation in travel time to nearest healthcare facility [58]. It would be possible to use this approach to model patient travel disruption from flooding and thereby assess its impact on healthcare utilisation. Since key informants in our study noted the impact of flooding on non-resident healthcare staff’s ability to travel to facility workplaces or to deliver community-based services such as immunisation or post-natal home visits, these service delivery outcomes should also be considered in relation to healthcare staff travel disruption in future work. Healthcare staff at primary facilities have also reported that flooding disrupts their ability to refer patients to secondary care because of travel difficulties [56], so there would be potential for future studies to assess disruption to patient referrals resulting from flooding.

## Conclusions

Our study demonstrates how satellite-derived flood products can be combined with routine health management information systems data to quantify flooding’s impact on health and healthcare utilisation. Given that Ghana’s DHIMS2 health management information system is used in 60 countries worldwide, this approach is potentially internationally transferable. Such analyses could enable spatial targeting of flood mitigation or health system adaptation measures, such as use of temporary, more accessible locations for healthcare delivery during flooding. We recommend that communities and health system professionals should collaborate to plan spatially targeted adaptation, mitigation and resilience strategies that explicitly address population and workforce mobility issues. Quantification of flooding’s impact could strengthen the business case for investing in these measures. The qualitative component of our study highlights a need for flood exposure metrics that capture further pathways by which flooding disrupts healthcare delivery, apart from population exposure to flooding and inundation of health facilities. These metrics relate to disruption to referrals, outpatient travel to facilities, and staff travel to facilities and for community outreach and could be evaluated via future studies. District disaster response, health, and local government teams should monitor such metrics to mitigate disruptions to healthcare delivery.

## Supplementary Information

**Additional file 1.** Focus Group Discussion guide.

**Additional file 2.** Key Informant Interview guide.

**Additional file 3.** Trend of diarrhoea, outpatient attendance and floods.

## Data Availability

OpenStreetMap data are available from https://www.openstreetmap.org/. SRTM digital elevation model data are available from https://doi.org/10.5066/F7PR7TFT. Gridded population data are available from https://www.worldpop.org/doi/10.5258/SOTON/WP00098. NRT MODIS flood product data are available from https://floodmap.modaps.eosdis.nasa.gov/. CHIRPS rainfall data are available from: https://data.chc.ucsb.edu/products/CHIRPS-2.0/. The LandSat Global Surface Water data set can be downloaded from: https://global-surface-water.appspot.com/download. The Copernicus global land cover product is available from https://land.copernicus.eu/global/products/lc. Requests for outpatient attendance and monthly case count data should be directed to Ghana Health Services [website: https://www.ghs.gov.gh/, Postal Address: Ghana Health Service, PMB, Ministries, Accra, Ghana, email: info@ghs.gov.gh]. District Health Management Information Systems data are available on request, subject to approval, from Ghana Health Services.

## Abbreviations

AIC: Akaike Information Criterion
BIC: Bayesian Information Criterion
CHIRPS: Climate Hazards Group InfraRed Precipitation with Station
CHPS: Community Health Planning and Services
DHIMS: District Health Management Information Systems
FGD: Focus Group Discussion
GHS: Ghana Health Service
GSW: Global Surface Water
HRSL: High Resolution Settlement
KII: Key Informant Interview
MODIS: Moderate Resolution Imaging Spectroradiometer
NADMO: National Disaster Management Organisation
NRT: Near Real Time
NTLHRSL: Night Time Light-High Resolution Settlement Layer
OPD: Out-Patient Department
