# Supplementary material for: Dam-mediated flooding impact on outpatient attendance and diarrhoea cases in northern Ghana: a mixed methods study
Source: BMC Public Health. 2022 Nov 17;22:2108. doi: 10.1186/s12889-022-14568-w (PMC9670488; doi:10.1186/s12889-022-14568-w)
Supplement: Supplementary file 1 — Additional file 1. Focus Group Discussion guide. [file 12889_2022_14568_MOESM1_ESM.pdf]

Focus Group Discussion (FGD) Guide for community members

An assessment of flooding from dam releases and its impacts on diarrhoea disease and microbiological contamination of water sources in selected dryland areas in Northern Ghana.

Date: ___ / ____ / ___ **Translator / recorder name: ____________**

Note-taker: ______________ **Location: __________________**

Start time: _____: _____
End time: _____: _____

Number of male participants: _________

Number of female participants: ____________

Pseudonyms / IDs for participants and seating chart

[Introduce project objectives. Read Consent to participate among the group and seek consent to continue]

1. Please can you tell me how the flood has affected the people and properties in this community?
2. Has there been an outbreak of diseases or an increase in some particular diseases as a result of the flooding?
3. How are the situations of healthcare access during flooding?
4. How do people in this community normally seek treatment during flooding periods [Probe for whether local traditional treatment methods or orthodox methods are used to seek treatment]
5. What are some of the early warning information you get about the flood and from where and when do you receive such information?
6. What are some of the strategies or measures you put in place to wait for the floods? [Probe for]
   - I. Crops planting and livestock protection.
   - II. Storing essential items (food, water, seeds, firewood, stoves, matches, items destroyable by water).
7. What are some of the adaptation or coping strategies employed during flooding to mitigate the adverse effects? [Probe for]
   - I. Water and Food protection (source protection, changes in food consumption behavior)
   - II. Safety of family members (canoes for movements, stay at lower risk areas)
   - III. Measures to protect livestock, poultry, and other assets (feed, shelter/cage)
   - IV. Migration, involving Income-generation activities (IGA) (food for work, labor-intensive jobs in cities)
   - V. Land selling and other assets
   - VI. Borrowing of money.
   - VII. Others……..
8. What are some of the activities you carry out after the flooding to recover all damages and harms caused by the flood?
9. Are there any gender roles in the strategies to mitigate the flooding adverse effects?
10. What are some of the constraints which impedes the community from adapting/coping with flood impacts?
11. Any recommendation and final words about the flooding events in this community?
