# Supplementary material for: Dam-mediated flooding impact on outpatient attendance and diarrhoea cases in northern Ghana: a mixed methods study
Source: BMC Public Health. 2022 Nov 17;22:2108. doi: 10.1186/s12889-022-14568-w (PMC9670488; doi:10.1186/s12889-022-14568-w)
Supplement: Supplementary file 2 — Additional file 2. Key Informant Interview guide. [file 12889_2022_14568_MOESM2_ESM.pdf]

Key Informant Interviews Guide

An assessment of flooding from dam releases and its impacts on diarrhoea disease and microbiological contamination of water sources in selected dryland areas in Northern Ghana.

[Introduce project objectives. Read Consent to participate and seek consent to continue]

Date: ____ / ____ / ____ Location: ____________

Respondent name: _____________ Respondent ID: _____

Circle the appropriate respondent

Assembly Men/women	AM
Water Providers	WP
Property Owners (house/farms)	PO
Health Care Providers	HCP
NADMO Officers	NO
District Coordinating Director	DCD
Red Cross Officer	RCO

1. *(ALL)* - Please can you tell me some of the measures your sector/office has put in place to lessen the impact of floods on the community.
2. *(HCP)* - How do you sensitize the community members to build their resilience to health in relation to the flood?
3. (*HCP*) – Are there any key health-related issues observed in flooded communities over the past years?
4. *(HCP)* - What are some of the challenges during flooding with respect to healthcare access.
5. *(ALL)* - What are some of the emergency approaches/strategies your sector employs during the flood?
6. Please are there any gender roles associated with these strategies?
7. (*ALL*) - What are some of the constraints affecting your sector/office in deploying these strategies and measures?
8. (*ALL*) - What will be your recommendations to resolve these constraints?
9. (*ALL*) - What are some of the support your sector gives to the community members in building their capacity to cope and adapt to the flooding.
10. (*ALL*) - What strategies do you think should be done to improve community capacity in flood adaptation?
11. (*ALL*) - Please how does your office respond to damages, affected persons, and properties after the floods?
12. (*ALL*)- What will be your assessment of the community's capacity in terms of flood adaptation?
