# Supplementary material for: Dam-mediated flooding impact on outpatient attendance and diarrhoea cases in northern Ghana: a mixed methods study
Source: BMC Public Health. 2022 Nov 17;22:2108. doi: 10.1186/s12889-022-14568-w (PMC9670488; doi:10.1186/s12889-022-14568-w)

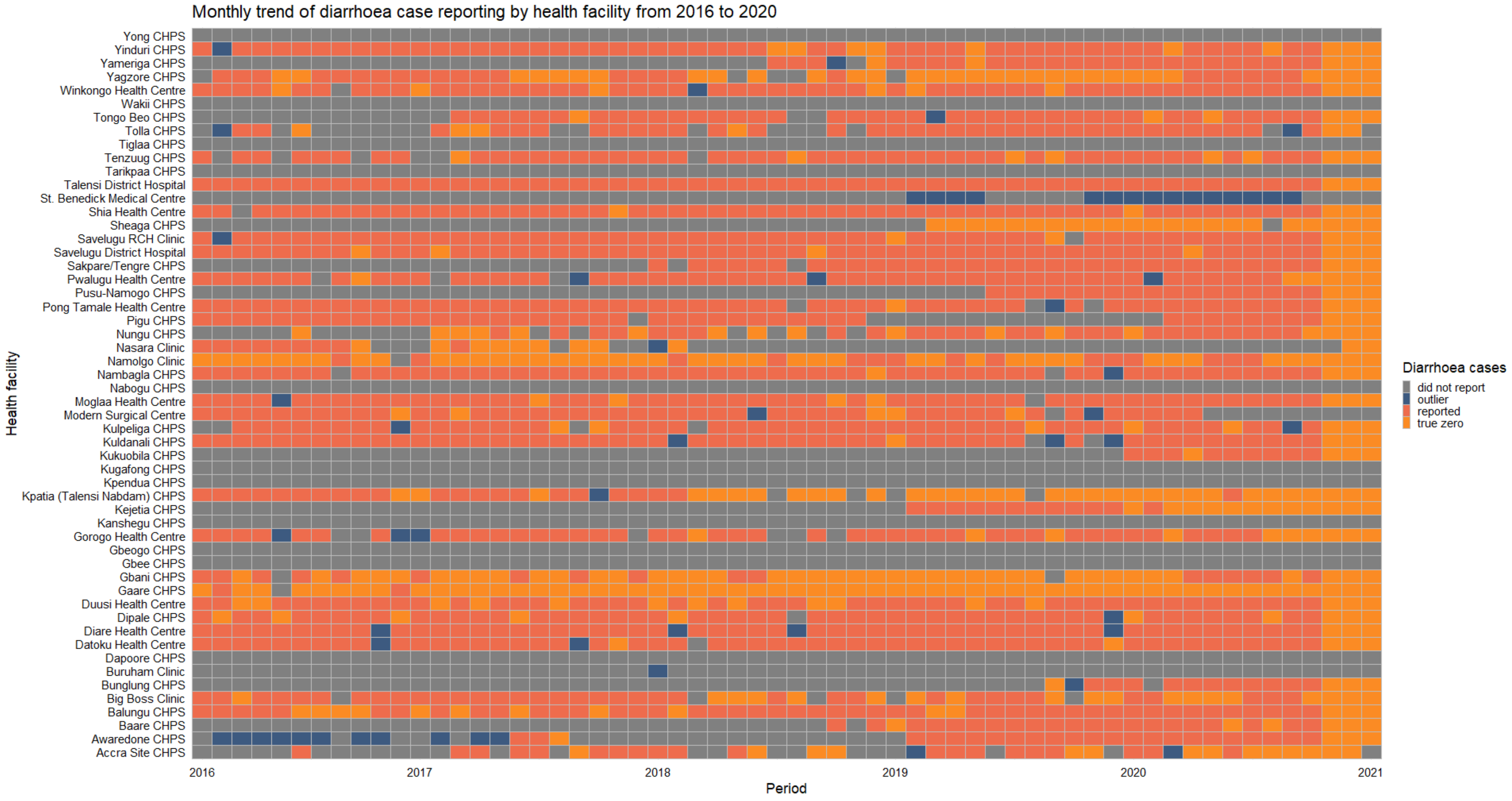
Monthly trend of diarrhoea case reporting by health facility from 2016 to 2020
Health facility
Yong CHPS
Yinduri CHPS
Yameriga CHPS
Yagzore CHPS
Winkongo Health Centre
Wakii CHPS
Tongo Beo CHPS
Tolla CHPS
Tiglaa CHPS
Tenzuug CHPS
Tarikpaa CHPS
Talensi District Hospital
St. Benedick Medical Centre
Shia Health Centre
Sheaga CHPS
Savelugu RCH Clinic
Savelugu District Hospital
Sakpare/Tengre CHPS
Pwalugu Health Centre
Pusu-Namogo CHPS
Pong Tamale Health Centre
Pigu CHPS
Nungu CHPS
Nasara Clinic
Namolgo Clinic
Nambagla CHPS
Nabogu CHPS
Moglaa Health Centre
Modern Surgical Centre
Kulpeliga CHPS
Kuldanali CHPS
Kukuobila CHPS
Kugafong CHPS
Kpendua CHPS
Kpatia (Talensi Nabdam) CHPS
Kejetia CHPS
Kanshegu CHPS
Gorogo Health Centre
Gbeogo CHPS
Gbee CHPS
Gbani CHPS
Gaare CHPS
Duusi Health Centre
Dipale CHPS
Diare Health Centre
Datoku Health Centre
Dapoore CHPS
Buruham Clinic
Bunglung CHPS
Big Boss Clinic
Balungu CHPS
Baare CHPS
Awaredone CHPS
Accra Site CHPS
2016
2017
2018
2019
2020
2021
Period
Diarrhoea cases
did not report
outlier
reported
true zero

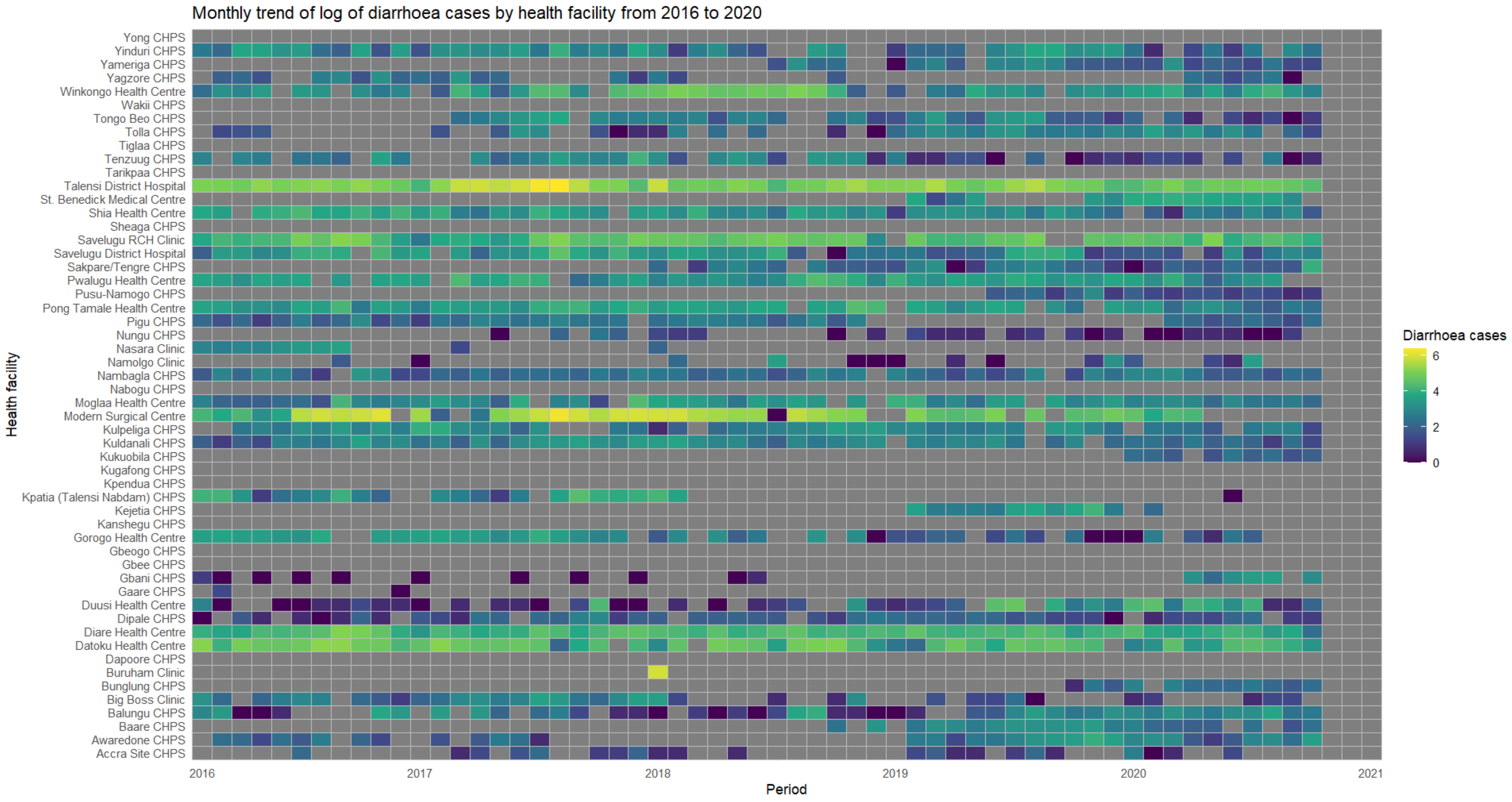

Monthly trend of log of diarrhoea cases by health facility from 2016 to 2020
Health facility
Yong CHPS
Yinduri CHPS
Yameriga CHPS
Yagzore CHPS
Winkongo Health Centre
Wakii CHPS
Tongo Beo CHPS
Tolla CHPS
Tiglaa CHPS
Tenzuug CHPS
Tarikpaa CHPS
Talensi District Hospital
St. Benedick Medical Centre
Shia Health Centre
Sheaga CHPS
Savelugu RCH Clinic
Savelugu District Hospital
Sakpare/Tengre CHPS
Pwalugu Health Centre
Pusu-Namogo CHPS
Pong Tamale Health Centre
Pigu CHPS
Nungu CHPS
Nasara Clinic
Namolgo Clinic
Nambagla CHPS
Nabogu CHPS
Moglaa Health Centre
Modern Surgical Centre
Kulpeliga CHPS
Kuldanali CHPS
Kukuobila CHPS
Kugafong CHPS
Kpendua CHPS
Kpatia (Talensi Nabdam) CHPS
Kejetia CHPS
Kanshegu CHPS
Gorogo Health Centre
Gbeogo CHPS
Gbee CHPS
Gbani CHPS
Gaare CHPS
Duusi Health Centre
Dipale CHPS
Diare Health Centre
Datoku Health Centre
Dapoore CHPS
Buruham Clinic
Bunglung CHPS
Big Boss Clinic
Balungu CHPS
Baare CHPS
Awaredone CHPS
Accra Site CHPS
2016
2017
2018
2019
2020
2021
Period
Diarrhoea cases
6
4
2
0

Monthly trend of log of outpatient attendance by health facility from 2016 to 2020

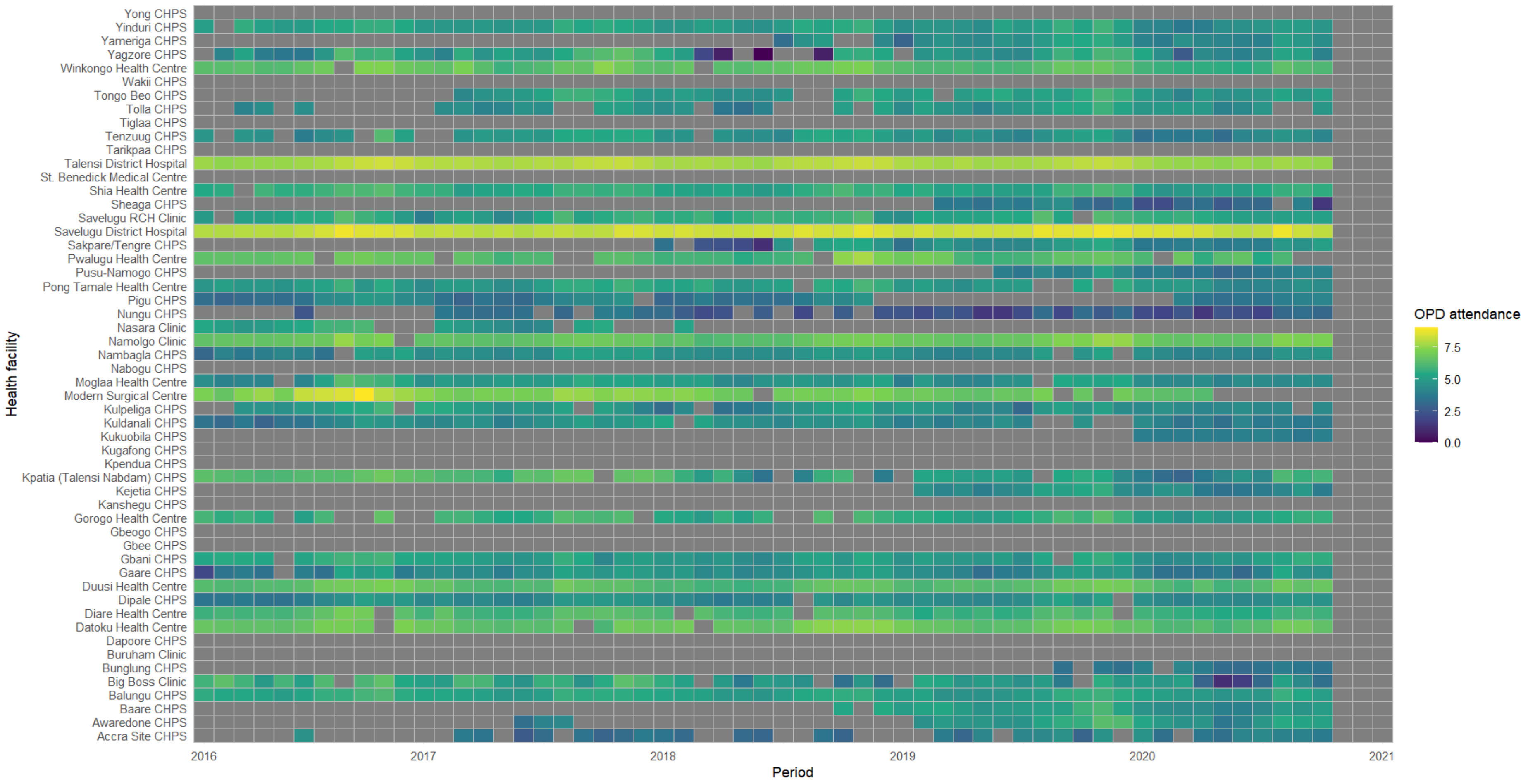

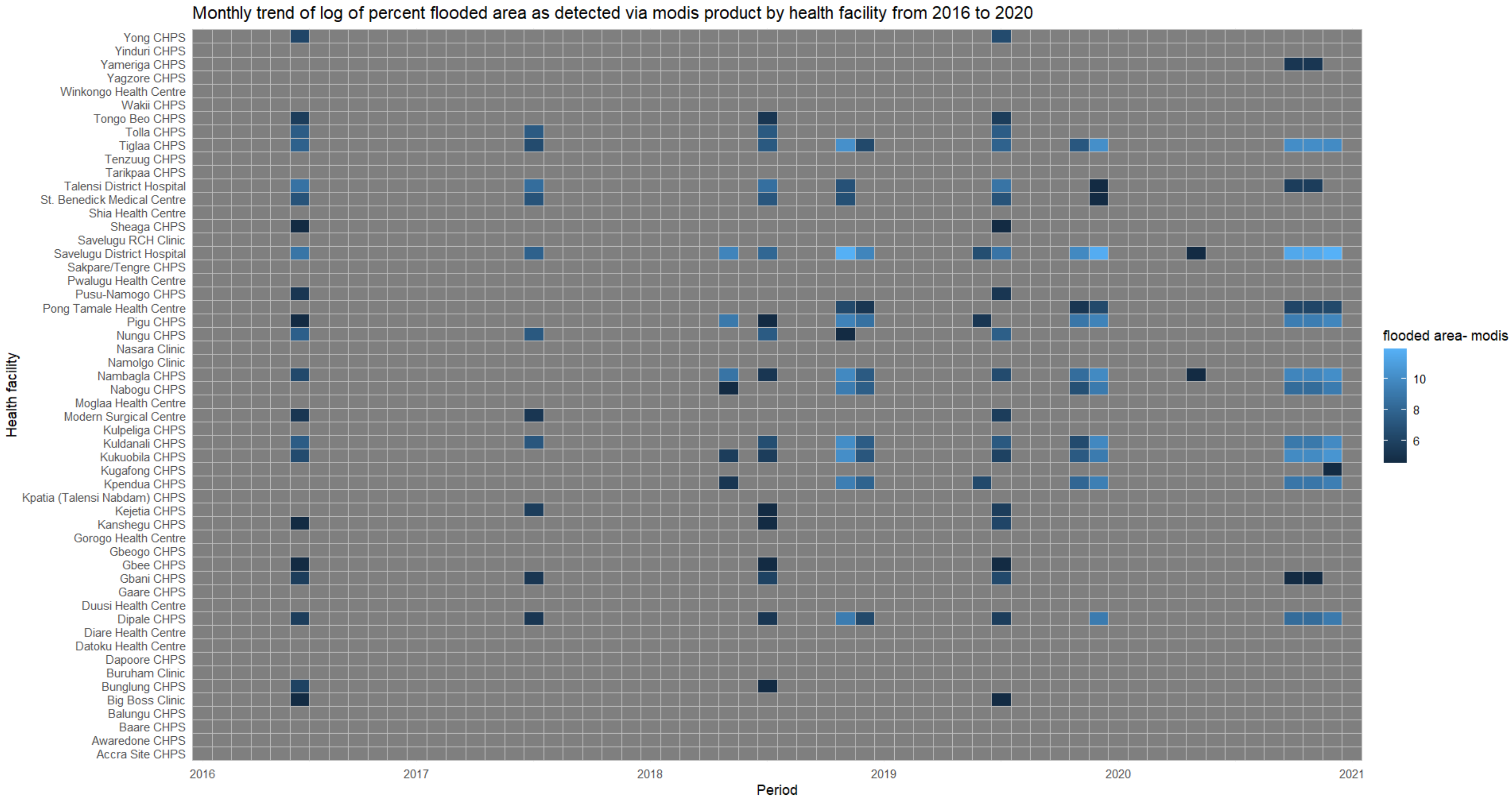
Monthly trend of log of percent flooded area as detected via modis product by health facility from 2016 to 2020
Health facility
Yong CHPS
Yinduri CHPS
Yameriga CHPS
Yagzore CHPS
Winkongo Health Centre
Wakii CHPS
Tongo Beo CHPS
Tolla CHPS
Tiglaa CHPS
Tenzuug CHPS
Tarikpaa CHPS
Talensi District Hospital
St. Benedick Medical Centre
Shia Health Centre
Sheaga CHPS
Savelugu RCH Clinic
Savelugu District Hospital
Sakpare/Tengre CHPS
Pwalugu Health Centre
Pusu-Namogo CHPS
Pong Tamale Health Centre
Pigu CHPS
Nungu CHPS
Nasara Clinic
Namolgo Clinic
Nambagla CHPS
Nabogu CHPS
Moglaa Health Centre
Modern Surgical Centre
Kulpeliga CHPS
Kuldanali CHPS
Kukuobila CHPS
Kugafong CHPS
Kpendua CHPS
Kpatia (Talensi Nabdam) CHPS
Kejetia CHPS
Kanshegu CHPS
Gorogo Health Centre
Gbeogo CHPS
Gbee CHPS
Gbani CHPS
Gaare CHPS
Duusi Health Centre
Dipale CHPS
Diare Health Centre
Datoku Health Centre
Dapoore CHPS
Buruham Clinic
Bunglung CHPS
Big Boss Clinic
Balungu CHPS
Baare CHPS
Awaredone CHPS
Accra Site CHPS
2016
2017
2018
2019
2020
2021
Period
flooded area- modis
10
8
6

Monthly trend of log of percent flooded area as detected via landsat product by health facility from 2016 to 2020

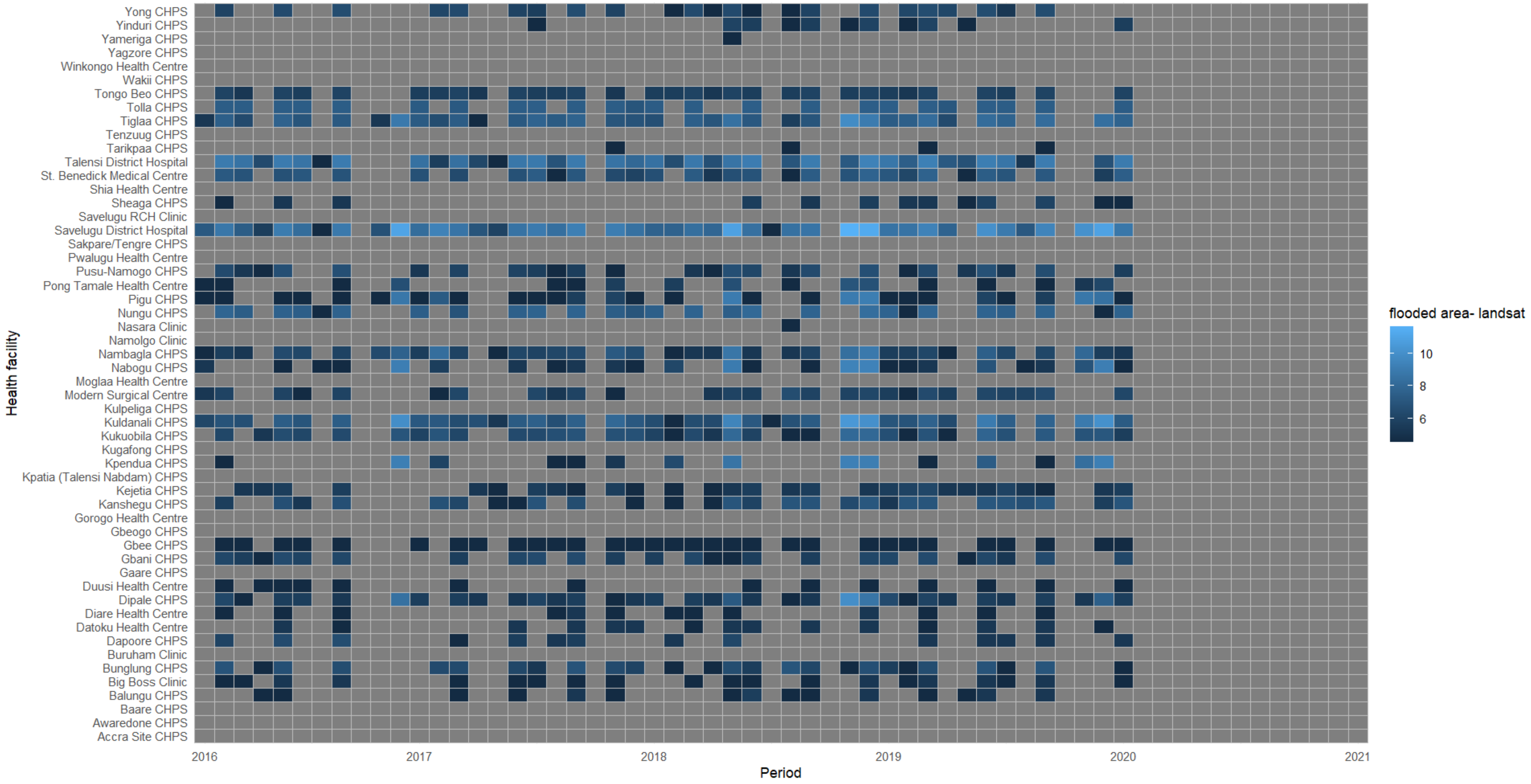

Supplement: Supplementary file 3 — Additional file 3. Trend of diarrhoea, outpatient attendance and floods. [file 12889_2022_14568_MOESM3_ESM.pdf]
